# High-efficiency and high-power rechargeable lithium–sulfur dioxide batteries exploiting conventional carbonate-based electrolytes

**DOI:** 10.1038/ncomms14989

**Published:** 2017-05-11

**Authors:** Hyeokjun Park, Hee-Dae Lim, Hyung-Kyu Lim, Won Mo Seong, Sehwan Moon, Youngmin Ko, Byungju Lee, Youngjoon Bae, Hyungjun Kim, Kisuk Kang

**Affiliations:** 1 Department of Materials Science and Engineering, Research Institute of Advanced Materials, Seoul National University, 1 Gwanak-ro, Gwanak-gu, Seoul 151-742, Republic of Korea; 2 Center for Nanoparticle Research at Institute for Basic Science (IBS), Seoul National University, 1 Gwanak-ro, Gwanak-gu, Seoul 151-742, Republic of Korea; 3 Graduate School of Energy Environment Water Sustainability (EEWS), Korea Advanced Institute of Science and Technology (KAIST), 291 Daehak-ro, Yuseong-gu, Daejeon 305-701, Republic of Korea

## Abstract

Shedding new light on conventional batteries sometimes inspires a chemistry adoptable for rechargeable batteries. Recently, the primary lithium-sulfur dioxide battery, which offers a high energy density and long shelf-life, is successfully renewed as a promising rechargeable system exhibiting small polarization and good reversibility. Here, we demonstrate for the first time that reversible operation of the lithium-sulfur dioxide battery is also possible by exploiting conventional carbonate-based electrolytes. Theoretical and experimental studies reveal that the sulfur dioxide electrochemistry is highly stable in carbonate-based electrolytes, enabling the reversible formation of lithium dithionite. The use of the carbonate-based electrolyte leads to a remarkable enhancement of power and reversibility; furthermore, the optimized lithium-sulfur dioxide battery with catalysts achieves outstanding cycle stability for over 450 cycles with 0.2 V polarization. This study highlights the potential promise of lithium-sulfur dioxide chemistry along with the viability of conventional carbonate-based electrolytes in metal-gas rechargeable systems.

## Abstract (web summary)

Commercial electrolytes for lithium ion batteries cannot be used for current metal-gas batteries due to the instability despite various merits. Here, the authors successfully exploit a widely used carbonate-based electrolyte to boost the electrochemical performance of lithium-sulfur dioxide batteries for the first time.

To satisfy the growing demand on energy storage devices for emerging electric vehicles and grid-scale energy storage markets, recent efforts have been devoted to exploring a new battery chemistry that can outperform the current state-of-the-art lithium ion batteries^1^,^2^,^3^. Revisiting the conventional primary batteries can offer insight for developing a novel rechargeable chemistry by taking advantage of the recent advances in the fundamental understanding of battery science^4^,^5^,^6^. One recent example is the development of rechargeable lithium-sulfur dioxide (Li-SO_2_) batteries. The primary Li-SO_2_ battery offers a high energy density in a wide operating temperature range with exceptionally long shelf-life and has thus been commercialized for military and aerospace applications since it was developed in the late 1960s (refs 7, 8, 9, 10). Recently, the Li-SO_2_ chemistry was revisited and proposed as a promising rechargeable battery chemistry^11^,^12^. Under an analogous cell configuration adopted from lithium-oxygen batteries, it has been demonstrated that a reversible electrochemical reaction between Li and SO_2_ is possible with the formation and decomposition of lithium dithionite (Li_2_S_2_O_4_). Similar to lithium-oxygen and lithium-sulfur batteries^6^, the absence of heavy transition metals in the redox reaction can result in a high energy density reaching 1,132 Wh kg^−1^ based on the mass of Li_2_S_2_O_4_. Moreover, the intrinsically smaller polarization and higher gas efficiency were observed to be advantageous compared with lithium-oxygen systems, making the Li-SO_2_ battery a potential metal-gas rechargeable battery chemistry.

Recent studies have revealed that the properties of the electrolyte in metal-gas batteries play a pivotal role in determining the nature of discharge products and the rechargeability of the system^13^,^14^,^15^,^16^,^17^,^18^,^19^. The discharge mechanisms and nature of discharge products such as their morphology and stability can be significantly altered by the properties of the electrolyte, such as donor numbers or dielectric constants^20^,^21^,^22^,^23^,^24^,^25^. The critical dependency on the electrolyte in the metal-gas system compared with conventional lithium/sodium ion batteries is most likely due to the generation of gas radicals, which are an important intermediate for the discharge reaction. Depending on the stability of the electrolyte, the gas radicals can react with the electrolyte solvent rather than the desirable alkali ions, such as lithium or sodium, which can form rechargeable discharge products^15^,^26^,^27^. Moreover, the stability of the intermediate alkali–radical complex is governed by the nature of the electrolyte and can thus alter the discharge paths^21^,^22^,^23^. In the early development of rechargeable lithium-oxygen or sodium-oxygen batteries, the use of carbonate-based electrolytes yielded side reaction products; thus, an appropriate charging process could not be achieved^14^,^15^,^19^. The organic carbonate was highly vulnerable to chemical attacks by the oxygen radicals generated during the discharge process^14^,^15^,^26^,^28^. This finding led to the overall perception that carbonate-based electrolytes cannot be considered for metal-air batteries. Nevertheless, carbonate-based electrolytes possess several benefits, including high ionic conductivity and wide electrochemical windows, which have made them a common electrolyte for the well-developed lithium/sodium ion batteries technology^29^,^30^. Moreover, the good lithium metal compatibility and chemical stability can be potential merits for lithium-air battery systems, which are expected to use lithium metal anodes and operate in an open system.

In this work, we evaluate the feasibility of implementing a conventional carbonate-based electrolyte in a Li-SO_2_ battery and investigate how the electrochemical properties are affected. Although the stability of SO_2_ gas radicals during the discharge process is unknown, we observe that the chemical reactivity of SO_2_^−^ towards organic carbonates is both thermodynamically and kinetically prohibited according to density functional theory (DFT) calculations. It is also experimentally verified that a Li-SO_2_ battery employing ethylene carbonate (EC) and dimethyl carbonate (DMC) as electrolytes can reversibly operate with the formation and decomposition of a Li_2_S_2_O_4_ discharge product. Furthermore, it is revealed that the power capability and cycling properties of the Li-SO_2_ batteries are remarkably improved compared with those using an ether-based electrolyte because of its higher ionic conductivity and better compatibility with the lithium metal anode. By introducing a soluble catalyst, cycling over 450 cycles is demonstrated with a high energy efficiency, exhibiting an overall polarization of 0.2 V during cycling, which has not yet been achieved in either lithium-oxygen or Li-SO_2_ batteries. This report is the first to demonstrate that conventional carbonate-based electrolytes can be successfully applied in rechargeable metal-gas systems, opening up a new avenue towards high-efficiency rechargeable metal-gas batteries.

## Results

### Theoretical investigation of Li-SO_2_ chemistry

The basic principle of Li-SO_2_ battery operation is based on the simple electrochemical reaction between Li and SO_2_ gas, whose general discharge reaction is^31^,^32^:


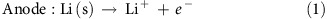



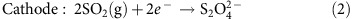



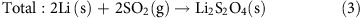



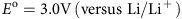


**Figure 1:**
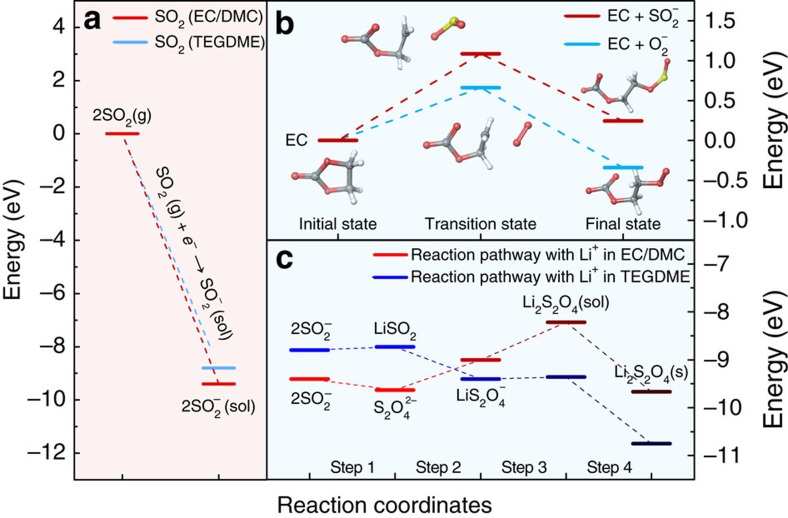
DFT calculation of the reaction chemistry of Li-SO_2_ batteries. (**a**) Energy diagrams for electrochemical reduction reaction of SO_2_ gas under EC/DMC and TEGDME. (**b**) Energy profiles for ICF of chemical EC decomposition reaction by O_2_^−^ (blue) and SO_2_^−^ (red). (**c**) Reaction pathway between SO_2_^−^ and lithium ions with corresponding energy profiles under EC/DMC (red) and TEGDME (blue).

In the cathode reaction, the SO_2_ collects the electron from the electrode and forms the intermediate dithionite ion (S_2_O_4_^2−^) before precipitating as solid Li_2_S_2_O_4_, the final discharge product. However, recent findings on O_2_^−^ in the lithium-oxygen chemistry suggest that the intermediate S_2_O_4_^2−^ may undergo chemical interactions with surrounding electrolyte molecules, which may lead to alternation in the discharge reaction path^21^,^22^. To investigate this early stage of the discharge reaction, we used DFT calculations coupled with the Poisson–Boltzmann (PB) solvation model to explore the reaction thermodynamics of Li-SO_2_ batteries. Moreover, similar calculations were performed under different electrolyte conditions to probe the effect of the surrounding electrolyte molecules on this discharge reaction. We selected two types of electrolyte: a conventional carbonate-based electrolyte (EC/DMC, 1:1 volume mixture) and ether-based electrolyte (tetraethylene glycol dimethyl ether, TEGDME), both of which have been widely used for lithium-ion and lithium-air rechargeable batteries^33^,^34^,^35^,^36^. Figure 1a shows the energy of the first electron transfer step starting from the SO_2_ molecule in a gas phase to SO_2_^−^ in the respective electrolyte solution. A slightly different energy trajectory of the electron transfer was observed in the two electrolyte systems, where the SO_2_^−^ in the EC/DMC is more stable by 0.30 eV than that in the TEGDME. The slightly different stabilization of the charged species is mainly attributed to the strong solvating ability of the carbonate-based electrolyte with the high dielectric constant (*ɛ*∼35)^20^,^37^.

Under normal operation conditions, it is expected that the electrochemically reduced SO_2_^−^ would react with lithium ions, leading to the formation of solid discharge products. However, the SO_2_^−^ in Li-SO_2_ cells may also undergo a chemical reaction with the carbonate-based electrolyte by nucleophilic attack, that is, electrolyte decomposition similar to the behaviour of O_2_^−^ in the electrolytes of lithium-oxygen batteries^13^,^26^. The plausibility of this chemical reaction can be determined by assessing the energetics of the initial complex formation (ICF) process involving the electrolyte molecule and SO_2_^−^20,26,27. Figure 1b presents the energy profile for the ICF processes involving EC molecules with the nucleophilic attack of SO_2_^−^ compared with that of the previously known case of O_2_^−^. Consistent with earlier theoretical^20^,^26^ and experimental studies^14^, the ICF of EC-O_2_ (blue dotted line) is a energetically downhill process with a moderate activation barrier, which indicates the instability of the carbonate electrolyte on exposure to O_2_^−^. However, it is noted that the ICF of EC-SO_2_ (red dotted line) is energetically unfavourable by 0.24 eV. Moreover, the activation energy that needs to be overcome is as high as 1.08 eV, indicating that it is also kinetically hindered. This finding implies that the electrochemically driven SO_2_^−^ molecule is likely to be stable in contact with the carbonate-based electrolyte without triggering severe degradation of the electrolyte.

The initial discharge process was investigated in further detail by evaluating the energies of each elementary reaction step from the SO_2_^−^ molecule to the final Li_2_S_2_O_4_ product; however, the detailed electrochemical mechanism of Li-SO_2_ batteries remains poorly understood to date. Figure 1c illustrates the most favourable discharge paths with each step denoted with energies in TEGDME (blue line) and EC/DMC (red line) electrolytes using the PB solvation model, where only the dielectric constant of the specific solvent is considered. Note that the dielectric constant of organic electrolytes can slightly alter with the addition of SO_2_ into the solvents as shown in Supplementary Fig. 1 and the measured dielectric constant of organic solutions dissolving SO_2_ was used for theoretical calculations in this study. Moreover, it should be noted that the energy profiles of elementary cathode reactions in Fig. 1c are described without consideration of the energetics of the anode reaction and the total reaction energetics including the anodic part are summarized in Supplementary Table 1. For TEGDME, which has a low dielectric constant (weak electrostatic interaction), the initial process proceeds with SO_2_^−^ combining with lithium ions, forming the neutral intermediate species of LiSO_2_. The additional chemical association of SO_2_^−^ and lithium ions to LiSO_2_ results in the formation of the final product of Li_2_S_2_O_4_ during the continuous downhill energy process. In contrast, for EC/DMC with a relatively high dielectric environment (strong electrostatic interaction), SO_2_^−^ is prone to undergo a dimerization reaction, forming S_2_O_4_^2−^ rather than a neutral species with lithium ions, which is a well-known chemical equilibrium of 2SO_2_^−^↔S_2_O_4_^2−^ in organic chemistry and biochemistry^38^,^39^,^40^. In the subsequent reaction steps, two lithium ions are associated with S_2_O_4_^2−^, forming LiS_2_O_4_^−^ and Li_2_S_2_O_4_, undergoing substantial uphill energy processes before the precipitation of the final product. The notably different initial discharge steps in the two electrolytes are attributed to their distinct solvating characters, where the high-dielectric solvent (EC/DMC) stabilizes the charged species such as S_2_O_4_^2−^ more effectively, preserving the strong solvation shell, and the low-dielectric solvent (TEGDME) fails to stabilize them, thus preferring to form a neutral species (LiSO_2_) in the reaction. This theoretical tendency is in accordance with the previous experimental findings that the chemical equilibrium constant of dimerization reaction to dithionite ion has a positive correlation with the dielectric constant of organic solvent media^40^. It is noteworthy that the previously proposed discharge mechanism of the primary Li-SO_2_ battery was based on the formation of S_2_O_4_^2−^ rather than SO_2_^−^ or LiSO_2_ (refs 32, 38). It is our speculation that this finding is most likely due to the use of an acetonitrile-based electrolyte in most primary Li-SO_2_ batteries^8^,^9^,^10^,^32^, which has a high dielectric constant (*ɛ*∼35.9)^41^,^42^ comparable to that of EC/DMC. Although the overall processes to attain the final product of Li_2_S_2_O_4_ are thermodynamically favourable with identical energy change of overall reactions in both electrolytes as shown in Supplementary Table 1, it should be noted that the energy profiles along the elementary reaction pathways for Li-SO_2_ batteries were significantly different depending on the type of electrolyte. One energy profile consisted of a monotonous downhill process (TEGDME), and the other consisted of a mixed uphill and downhill process involving a significant energy barrier (EC/DMC). This difference in the reaction energetics is expected to affect the nature of the formation of solid discharge products for the Li-SO_2_ battery depending on the type of electrolyte, which will be discussed more in the experimental section.

### Feasibility of Li-SO_2_ chemistry in carbonate electrolytes

**Figure 2:**
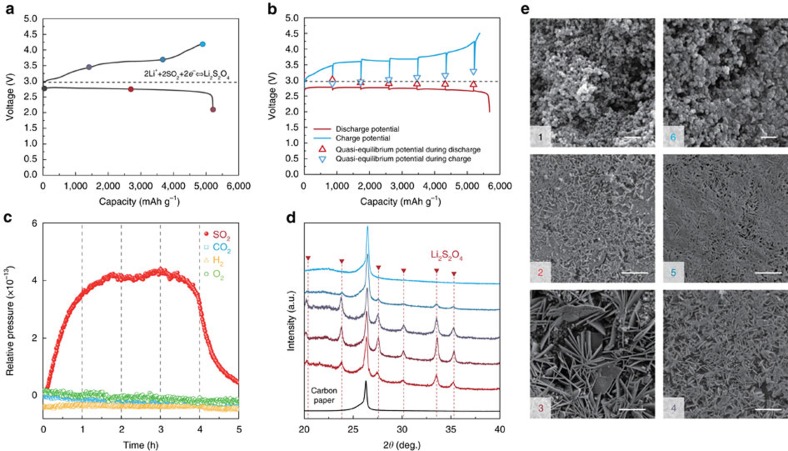
Reversible chemistry of Li-SO_2_ batteries under carbonate-based electrolyte. (**a**) Galvanostatic discharge/charge profile of Li-SO_2_ cell at a current density of 0.2 mA cm^−2^. (**b**) Galvanostatic intermittent titration technique (GITT) analysis result of Li-SO_2_ cell. The specific capacity was normalized by the mass of carbon loading whose average mass in a gas electrode was ∼0.8±0.1 mg. (**c**) *In situ* gas analysis during charge process of the Li-SO_2_ cell by DEMS. (**d**) *Ex situ* X-ray diffraction spectra of gas electrodes for Li-SO_2_ cells. (**e**) Corresponding *ex situ* SEM images of the gas electrodes of Li-SO_2_ cells (scale bar, 300 nm; scale bar, 5 μm (2–5)).

Inspired by the theoretical findings, we constructed Li-SO_2_ cells using the conventional carbonate electrolyte EC/DMC (1:1 volume ratio with 1 M lithium hexafluorophosphate) and examined the stability of the rechargeable Li-SO_2_ chemistry. Figure 2a presents the galvanostatic voltage profile during the first cycle of the Li-SO_2_ cell. The electrochemical profile at 0.2 mA cm^−2^ resembles the typical profile of the Li-SO_2_ cells using an ether-based electrolyte in a previous study^12^. To confirm the reversible electrochemical reactions, we performed several analyses of Li-SO_2_ cells using the galvanostatic intermittent titration technique, differential electrochemical mass spectroscopy (DEMS), X-ray diffraction and scanning electron microscopy (SEM). Figure 2b indicates that the equilibrium potentials measured by galvanostatic intermittent titration technique are in an agreement with the thermodynamic potential of Li_2_S_2_O_4_ (∼3 V)^43^, which supports the idea that the main reaction involves the formation and decomposition of Li_2_S_2_O_4_. Furthermore, the DEMS results in Fig. 2c indicate that the SO_2_ gas was solely detected without the evolution of other gases during the entire charge process, demonstrating the reversible and stable charge reaction occurring in the Li-SO_2_ cell. Considering that the oxygen evolution during the charging of conventional lithium-oxygen cells is typically accompanied by the release of considerable amounts of carbon dioxide due to electrolyte decomposition^15^,^17^ and carbon deterioration^44^,^45^, the absence of carbon dioxide in this experiment supports the idea that the EC/DMC electrolyte as well as the carbon electrode used for Li-SO_2_ cells remain stable during the cell operation. In addition to the evidence on a gas phase evolution of SO_2_ through *in situ* gas analyses, the characterization of electrolytes after charge of the Li-SO_2_ cells with ultraviolet –visible spectroscopy in Supplementary Fig. 2 clearly confirms the reversible evolution of SO_2_ from the electrolyte solution^46^,^47^.

To further verify the electrochemical reaction, we carefully performed *ex situ* analyses on the gas electrodes of Li-SO_2_ cells at different states of charge or discharge, as shown in Fig. 2a. The *ex situ* X-ray diffraction spectra in Fig. 2d reveal that characteristic peaks of Li_2_S_2_O_4_ appear and grow during discharge without any notable by-products, followed by the reduction of these peaks during the charge and their complete disappearance after the end of the charge^31^,^48^. These results evidently confirm that the reversible formation and decomposition of Li_2_S_2_O_4_ is the major electrochemical reaction occurring in the Li-SO_2_ system using an EC/DMC electrolyte, which is consistent with the DFT calculations. In addition, the formation and decomposition of Li_2_S_2_O_4_ can be directly probed by tracking the morphological evolution on the electrode in the *ex situ* SEM images in Fig. 2e. It is apparent that two-dimensional plates begin to appear on the carbon gas electrode on discharge and grow up to ∼5 μm in size, covering all the carbon surfaces at the end of discharge. On the charge process, Li_2_S_2_O_4_ gradually disappears; at the end of charge to 4.2 V, no micron plate was observed in the electrode, and the porous structure of the gas electrode was well recovered to its pristine state, which is in a good agreement with the X-ray diffraction results. Note that the morphological feature of the discharge product is slightly different from that of the Li_2_S_2_O_4_ formed using the TEGDME electrolyte in our previous study^12^. As carefully compared in Supplementary Fig. 3, the Li_2_S_2_O_4_ initially forms needle-like precipitates and grows into numerous nanoribbons for the TEGDME electrolyte, in contrast to the micron-sized Li_2_S_2_O_4_ plate in the EC/DMC electrolyte. Interestingly, the morphology of discharge products has recently been regarded as an important clue to understanding the discharge mechanism of metal-oxygen batteries^21^,^22^,^23^,^49^. In the lithium-oxygen battery system, for instance, highly solvating electrolytes with a high donor number or solvating additives promote the nucleation and growth of the crystalline toroidal Li_2_O_2_ with a typically large particle size by driving the solution-mediated process, whereas electrolytes with low donor numbers tend to form film-like discharge products on the surface of the electrode^21^,^22^,^50^. According to the reaction pathways examined by the DFT calculations in Fig. 1c, it is believed that the intermediate energy uphill processes in the EC/DMC electrolyte would play an important role in governing the nucleation of solid precipitates because of the critical energy barrier, in contrast to the case of TEGDME, where there is no energy barrier for the discharge process. Because the number of nuclei is generally inversely proportional to the nucleation energy barriers, we presumed that a small number of nuclei generated under highly solvating EC/DMC electrolyte yield to form the relatively well-grown micron-sized discharge products of Li_2_S_2_O_4_. Further study must be performed to understand the relationship between the discharge mechanism and the feature of the discharge products in the Li-SO_2_ system.

### Performance of Li-SO_2_ cells using carbonate electrolytes

**Figure 3:**
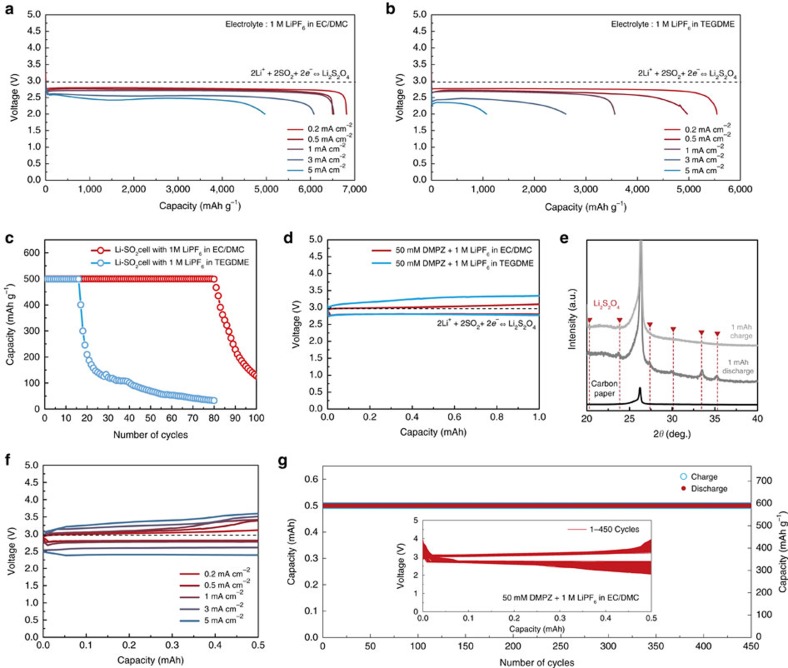
Electrochemical performance of Li-SO_2_ batteries. (**a**,**b**) Discharge rate capability of Li-SO_2_ cell: (**a**) with carbonate electrolyte, (**b**) with ether electrolyte and (**c**) cycle properties of Li-SO_2_ cells at 0.2 mA cm^−2^. (**d**) Electrochemical profiles of Li-SO_2_ cells with a soluble catalyst. (**e**) X-ray diffraction spectra of discharged and recharged gas electrode of Li-SO_2_ cell with soluble catalyst. (**f**,**g**) Electrochemical performance of Li-SO_2_ cells with soluble catalyst containing carbonate-based electrolyte: (**f**) power capability of the cells under limited capacity of 0.5 mAh, (**g**) cyclability of the cell at 1 mA cm^−2^. (inset: electrochemical profiles during 450 cycles.) The specific capacity was normalized by the mass of carbon loading whose average mass in a gas electrode was ∼0.8±0.1 mg.

Having confirmed the reversible Li_2_S_2_O_4_ formation in the carbonate-based electrolyte, the electrochemical properties of Li-SO_2_ cells were comparatively investigated in EC/DMC and TEGDME electrolytes. Figure 3a,b compare the power capability of Li-SO_2_ cells under current rates ranging from 0.2 to 5.0 mA cm^−2^ during discharge. Interestingly, a significantly higher rate capability is achievable with the cell employing the EC/DMC electrolyte for an identical cell configuration. Although similar discharge capacities are delivered at a low current rate of 0.2 mA cm^−2^ for the two cases, the cell with EC/DMC is capable of delivering more than 70% of the initial capacity even at 25 times higher current density; in contrast, the cell with TEGDME exhibits a negligible capacity at the same current rate. It is speculated that the facile ion transport in EC/DMC, which exhibits ∼4 times higher ionic conductivity than TEGDME, as shown in Supplementary Table 2, contributes to the high rate capability of the Li-SO_2_ cell. To verify the reversible Li_2_S_2_O_4_ formation/decomposition in such a high rate operation, the *ex situ* analyses were performed again under the condition shown in Supplementary Fig. 4, which revealed identical results regardless of the applied current densities. In Fig. 3c, the cycling properties of Li-SO_2_ cells were comparatively investigated at a constant rate of 0.2 mA cm^−2^. The Li-SO_2_ cell with EC/DMC stably operated over 80 cycles, whereas that with the TEGDME electrolyte maintained ∼20 cycles, which is consistent with the previous report^12^. Moreover, better cycle stability of the cell with EC/DMC was again confirmed with the larger capacity utilization of 1,000 mAh g^−1^, as shown in Supplementary Fig. 5. Note that no special treatment such as nanoscale gas electrode design or the use of a catalyst was applied during the test, under which conventional lithium-oxygen or sodium-oxygen batteries would exhibit significantly less cycle stability^51^,^52^,^53^,^54^. Even at a higher current density of 1 mA cm^−2^, the Li-SO_2_ cell with EC/DMC could sustain a high cycle stability of ∼50 cycles, as observed in Supplementary Fig. 6, supporting the idea that simply replacing the electrolyte could markedly enhance the cycling properties of Li-SO_2_ cells. The superior cycling performance of the Li-SO_2_ cell with EC/DMC is attributed to the stability of the carbonate-based electrolyte in the presence of the strongly solvated intermediate SO_2_^−^ product and the better chemical compatibility with the lithium anode, which will be discussed later.

To examine the practical viability of the Li-SO_2_ cells, it was attempted to further enhance the energy efficiency using an appropriate catalyst to promote the charging reaction. A soluble catalyst of 5,10-dimethylphenazine (DMPZ) was introduced into the electrolyte to decrease the charge polarization, which was recently reported as an efficient soluble catalyst for lithium-oxygen batteries^55^. Figure 3d presents the characteristic discharge/charge profiles with the DMPZ catalysts for the two Li-SO_2_ cells, which reveals substantial reduction in the charge overpotential. The charging voltage, that is, the oxidation potential of DMPZ, in the TEGDME electrolyte was analogous to that in our previous study in lithium-oxygen batteries^55^. However, surprisingly, the cell with the DMPZ catalyst in the EC/DMC electrolyte could be recharged at a voltage plateau of ∼3.0 V, which is almost identical to the thermodynamic potential of Li_2_S_2_O_4_. Thus, the overall polarization of the cells was only 0.2 V, resulting in an energy efficiency of ∼93.3%, one of the highest values accomplished with lithium-gas-type batteries. This finding indicates that DMPZ is not only capable of chemically decomposing Li_2_S_2_O_4_, similar to the case of Li_2_O_2_ in lithium-oxygen batteries, but also enables much a higher charging efficiency in the carbonate-based electrolyte. To confirm this unexpected dependency of the redox potential of DMPZ on the electrolyte species, the cyclic voltammetry test and galvanostatic charging in the inert atmosphere were performed again for the DMPZ dissolved in each electrolyte with the three-electrode configuration, as shown in Supplementary Fig. 7. DMPZ consistently exhibited a lower oxidation potential in EC/DMC than in TEGDME by ∼0.2 V. The lower oxidation potential of DMPZ under the carbonate electrolyte might be attributed to the strong stabilization effect on the charged species of DMPZ^+^ due to the highly solvating environment offered by the carbonate electrolytes. The alternation in the redox potential of soluble catalysts depending on the electrolyte media was also observed in a recent study, where the redox potential of a LiI catalyst was notably different in dimethoxyethane than in TEGDME^56^. *Ex situ* X-ray diffraction analysis confirmed that the catalytic activity of DMPZ in decomposing Li_2_S_2_O_4_ could be maintained even with the lower redox potential of DMPZ in EC/DMC. Figure 3e shows that characteristic diffraction patterns of Li_2_S_2_O_4_ were observed in the discharged electrode but disappeared after the charging, indicating the effective decomposition of the discharge product. Supplementary Fig. 8 further confirms the charging reaction based on the decomposition of Li_2_S_2_O_4_ by the DMPZ catalyst through X-ray photoelectron spectroscopy (XPS) and *in situ* gas analysis. On charging of the cell, the XPS signature of Li_2_S_2_O_4_ gradually fades away, which is accompanied by the evolution of SO_2_ without any other detectable gases, implying the efficient catalytic behaviour of DMPZ for Li-SO_2_ cells.

We further investigated the electrochemical performance of the Li-SO_2_ cell employing the carbonate electrolyte with the DMPZ catalyst. Figure 3f shows the power capability of the cell for current densities ranging from 0.2 to 5.0 mA cm^−2^ under a controlled capacity of 0.5 mAh. Although the overall polarization systematically increased as the applied current increased, the charge processes of all the cells could be performed below 4 V without exceeding the voltage limit, demonstrating the fast kinetics of the Li_2_S_2_O_4_ formation and decomposition aided by DMPZ. It was observed that the efficient catalytic activity of the DMPZ could also lead to a remarkable enhancement in the cycling performance of Li-SO_2_ cells. Figure 3g shows that the Li-SO_2_ cells employing EC/DMC with the DMPZ catalyst exhibit superior cycle stability of more than 450 cycles of 0.5 mAh, which has rarely been recorded for lithium-oxygen batteries with such a large absolute capacity. During 450 cycles of the Li-SO_2_ cells, the charging overpotential was only slightly increased, maintaining the high energy efficiency of the cell, as observed in the inset of Fig. 3g. This finding indicates that the catalytic activity of DMPZ is stably maintained and not consumed during the cell operations. All the electrochemical results support the idea that a battery with superior power, efficiency and reversibility is achievable using the Li-SO_2_ chemistry by employing a carbonate-based electrolyte and soluble catalyst.

## Discussion

**Figure 4:**
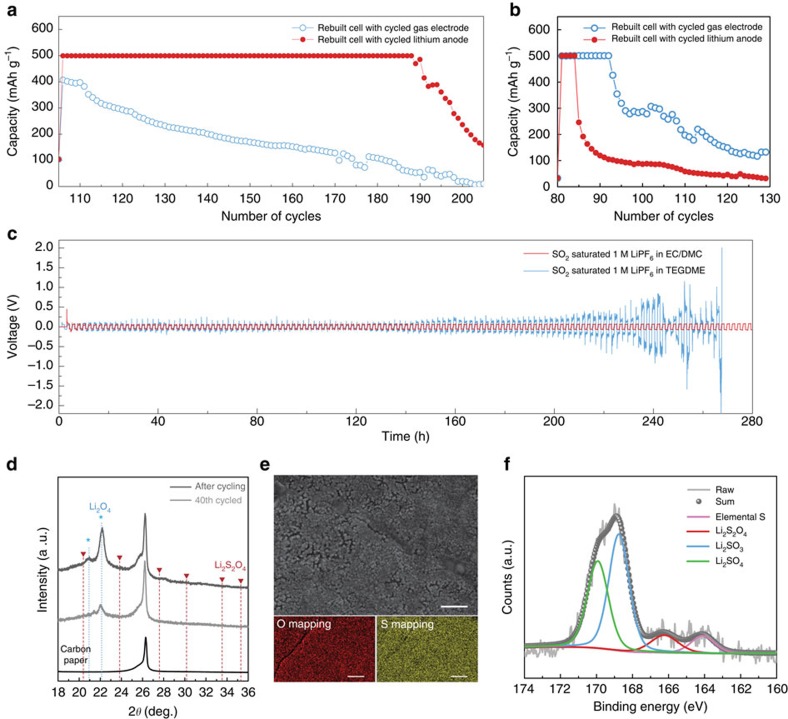
Investigation of cycle degradation of Li-SO_2_ batteries. (**a**,**b**) Cycle properties of rebuilt cells with cycled gas electrode or cycled lithium anode: (**a**) under carbonate electrolyte and (**b**) under ether electrolyte. The specific capacity was normalized by the mass of carbon loading whose average mass in a gas electrode was ∼0.8±0.1 mg. (**c**) Lithium symmetric cell test at a current density of 1 mA cm^−2^ with SO_2_-saturated EC/DMC (red) and TEGDME (blue). (**d**) X-ray diffraction spectra of gas electrode of Li-SO_2_ cells at the middle and end of cycles. (**e**) SEM images (scale bar, 500 nm) of gas electrode at the end of cycle and elemental mapping by energy-dispersive spectroscopy (scale bar, 50 μm). (**f**) XPS 2*p* spectra for gas electrode after cycling of Li-SO_2_ cell.

Despite the impressive cycle properties achieved with the catalyst, the origin of the cycle degradation should be understood for further development of Li-SO_2_ batteries. Given the improved cycle stability of the cell using the EC/DMC electrolyte, we attempted to comparatively elucidate how the different electrolytes affect the cycling performance by probing the respective degradation of the carbon gas electrode and lithium metal anode in addition to the electrolyte stability itself^17^,^57^. After the cell degradation in Fig. 3c, the cells were disassembled and each electrode was collected; the cells were then rebuilt with a fresh counter electrode and new electrolyte. Figure 4a compares the cycling properties of two rebuilt cells based on EC/DMC: one with the cycled lithium anode (red) and the other with the cycled gas electrode (blue). Interestingly, the Li-SO_2_ cell with the cycled lithium metal anode could reproduce the original cyclability of ∼80 cycles, which suggests that the lithium metal cycled in the EC/DMC electrolyte was not significantly degraded. Note that the experiment was performed without DMPZ catalysts; thus, the original cycle life was ∼80 cycles in Fig. 3c. However, the rebuilt cell with the cycled gas electrode could not cycle stably. This finding clearly indicates that the degradation of the gas electrode is the main cause of the overall cycle deterioration in Li-SO_2_ cells using the EC/DMC electrolyte. In contrast, the opposite result was observed for the same experiments conducted for the cells employing the TEGDME electrolyte, as shown in Fig. 4b. The rebuilt cell with the cycled gas-electrode exhibited similar cycle properties of ∼20 reversible cycles as the original cell in Fig. 3c. However, the cell with the cycled lithium metal anode could not stably function, as observed in Fig. 4b, indicating that the degraded lithium metal anode was the main cause of the rapid cycle deterioration of the Li-SO_2_ cells using the TEGDME electrolyte. The severe degradation of lithium metal was again supported by an experiment in which the lithium metal anode was replaced multiple times, which led to a comparable cycling property as that of the rebuilt cell using the cycled gas electrode, as shown in Supplementary Fig. 9. To confirm the higher stability of the lithium metal anode in EC/DMC, a lithium metal symmetric cell was constructed, as shown in Fig. 4c. The usage of the carbonate electrolyte led to a much smaller polarization and longer operating time than those using the ether electrolyte, which is in a good agreement with previous studies^58^,^59^. This finding supports the idea that the better cycling properties of the Li-SO_2_ cells employing EC/DMC are partly attributable to the highly stable lithium metal interfaces because of better lithium metal compatibility with the carbonate electrolyte.

For a more comprehensive understanding of the cycle degradation of Li-SO_2_ cells employing the carbonate electrolyte, we examined the carbon gas electrode after the cycling. The X-ray diffraction patterns in Fig. 4d reveal that after charging, the Li_2_S_2_O_4_ discharge product was hardly detectable; however, the characteristic peak of Li_2_SO_4_ began to appear appreciably even after 40 cycles, and a substantial amount of Li_2_SO_4_ by-products are detected at the end of the cycles. Although the expected discharge product, Li_2_S_2_O_4_, was clearly decomposed in the cycled cathodes, it is speculated that the gradual deposition of the insulating by-products on the carbon gas electrode would have a negative effect on the cycling behaviour of Li-SO_2_ cells. Moreover, examination of the morphology of the cycled gas electrodes clearly revealed that all the active pores of the gas electrodes were mostly blocked, as observed in Fig. 4e. Energy-dispersive spectroscopy analysis revealed that the densely clogged pores of the cycled gas electrode were mainly composed of sulfur and oxygen. The gradual deposition of insulating by-products results in the significant increase of total impedances of Li-SO_2_ cells, which is confirmed through the electrochemical impedance spectroscopy analyses with cycling the cells as shown in Supplementary Fig. 10. Consequently, the accumulation of inactive and insulating by-products on the pores of the gas electrode would restrict the active reaction sites and the transport of reactants, including lithium ions and SO_2_ gas, finally resulting in the cell failures. In previous studies on primary Li-SO_2_ batteries, the formation of such by-products has generally been attributed to the self-decomposition of Li_2_S_2_O_4_ due to its thermodynamic instability^11^,^48^,^60^. According to the XPS analysis of the surface of the cycled cathodes in Fig. 4f, four different oxidation states of sulfur were detected, including the residual discharge product, Li_2_S_2_O_4_, at 166.5 eV. The two major peaks at 168.7 and 169.8 eV are assigned to the sulfur from Li_2_SO_3_ and Li_2_SO_4_, respectively, which is consistent with our previous study.^12^ The presence of the Li_2_SO_4_ by-product corresponds well to the X-ray diffraction result in Fig. 4d. Note that a trace amount of elemental sulfur (164.1 eV) was detected in the XPS spectra, which hints at the formation mechanism of Li_2_SO_4_. According to the self-decomposition of Li_2_S_2_O_4_, which can occur spontaneously, as indicated by the DFT calculations in Supplementary Table 3, it should accompany the generation of elemental sulfur, that is, Li_2_S_2_O_4_ (s)→Li_2_SO_4_ (s)+S (s). The presence of both elemental sulfur and Li_2_SO_4_ in the cycled gas electrode strongly suggests that the self-decomposition of the discharge product can cause deterioration of the cycle performance. Conclusively, a strategy for improving the stability of discharge products and suppressing the formation of by-products should be further explored to develop a better-performing Li-SO_2_ battery.

We successfully employed a conventional carbonate-based electrolyte in rechargeable Li-SO_2_ batteries and validated the feasibility of the system through combined theoretical and experimental verifications. The chemical stability of the carbonate electrolyte against the reduced form of SO_2_ allowed Li-SO_2_ cells to be reversibly operated, unlike the conventional lithium-oxygen systems. The high ionic conductivity and chemical compatibility with the lithium metal anode led to a remarkable improvement of the Li-SO_2_ cell performances, including the power capability and cycle stability. Furthermore, the application of a DMPZ catalyst yielded one of the highest efficiencies (∼93.3%) and reversibilities (450 cycles) reported for metal-gas systems to date. Towards the realization of a practical rechargeable Li-SO_2_ battery system, several issues still need to be addressed, including the lack of fundamental understanding and safety issues regarding the use of a toxic gas. In this regards, more quantifiable characterization techniques inclusive of pressure monitoring^61^,^62^ and chemical titration methodology^22^,^63^, currently introduced in the research of metal-air batteries, should be considered for the elucidation of precise gas efficiency or side-reaction mechanism of the Li-SO_2_ chemistry in following studies. In addition, taking advantage of the commercialized primary Li-SO_2_ battery technology, closed-type pressurized systems could be one of the practically approachable models for the safe Li-SO_2_ secondary battery with potential merits of the enhanced operation voltage and the reversibility obtained in our preliminary experiments as described in Supplementary Fig. 11 and Supplementary Note 1. Nevertheless, this study offers insights to the metal-air battery community regarding the importance of the electrolyte and its compatibility with the lithium or sodium metal electrode considering that the exceptionally high theoretical capacity of lithium/sodium-oxygen batteries is partly attributed to the use of a metallic lithium or sodium electrode. Thus, more studies should focus on how to rationally control the interaction between the electrolyte and metal anodes in metal-oxygen batteries. We hope that this report will pave the way for a new field of Li-SO_2_ batteries as a promising next-generation battery system and spur vigorous discussions in the search for a robust electrolyte in the metal-air battery community.

## Methods

### Computational details

DFT calculations were performed using the Jaguar 8.9 software^64^ for molecular reaction energies under the PB implicit solvation condition. We used the exchange-correlation functional of B3LYP^65^,^66^ along with the Pople 6-311++G** basis set^67^. The ground electronic and geometric structures for molecular reaction intermediates were fully optimized for both gas and solution phases. Single-point solution phase calculations without relaxing the gas phase structure were conducted only for the transition state obtained from a simple quasi-Newton method that searches for the transition state nearest to the input guessed geometry. The initial guess for the transition state search was obtained by scanning the most unstable geometry along the expected reaction coordinates, and the obtained transition states were validated by checking the number of imaginary frequency from vibrational modes. We also used the Vienna *Ab initio* Simulation Package^68^ for the calculations of the cohesive energy of crystal structures with the exchange-correlation function of Perdew–Burke–Ernzerhof^69^. The electron–ion interaction was considered in the form of the projector augmented wave method with a plane wave up to an energy of 400 eV. Gamma-centred *k*-point grids of 10 × 10 × 10 for lithium metal, 5 × 5 × 5 for Li_2_S_2_O_4_ and 4 × 6 × 4 for Li_2_SO_4_ were generated. The ground electronic and geometric structures were fully optimized for the crystal and corresponding formula unit molecule for each crystal structure. Further details including solvent parameters (Supplementary Table 4) and hypothetical crystal structure (Supplementary Fig. 12) are discussed in Supplementary Information.

### Preparation and assembly of Li-SO_2_ cells

For the preparation of carbon paste, Ketjen black carbon (EC 600JD; Ilshin Chemtech) was dispersed with polytetrafluoroethylene (60 wt% dispersion in H_2_O) binder in a mass ratio of 8:2 into a solution of isopropanol (>99.7%; Sigma-Aldrich) and *N*-methyl-2-pyrrolidone (99.5%, anhydrous; Sigma-Aldrich) with a volume ratio of 1:1. The carbon gas electrode was fabricated by casting the carbon paste on the carbon paper current collectors (TGP-H030; Toray, Japan) and dried overnight at 120 °C to evaporate the solvent and residual water. The average loading mass of the Ketjen black electrodes in a 1/2-inch diameter was ∼0.8±0.1 mg. Electrolytes of 1 M lithium hexafluorophosphate (LiPF_6_) dissolved in EC/DMC 1:1 vol% or TEGDME with water contents less than 20 p.p.m. measured by Karl-Fisher titration were used. The Li-SO_2_ cell was assembled using a Swagelok-type cell in a sequence of lithium metal (3/8-inch diameter), two sheets of Celgard 2400 separators (1/2-inch diameter) and the prepared carbon electrode (1/2-inch diameter) in an argon-filled glove box (O_2_ level <1 p.p.m. and H_2_O level <1 p.p.m.). The amount of electrolyte was 200 μl. For the electrolytes employing the soluble catalyst, 50 mM concentration of DMPZ was added to the prepared electrolytes. The gas electrode of individual cells was open to the SO_2_ gas after the cell assembly and stabilized during a relaxation time of 0.5 h before the cell tests.

### Characterization of Li-SO_2_ cells

All the electrochemical tests for the Li-SO_2_ cells were performed using a potentiogalvanostat (WBCS 3000; WonA Tech, Korea) between 2.0 and 4.2 V at room temperature. For the lithium symmetric cell tests, a coin-type cell CR2032 was assembled with 1/2-inch diameter lithium foils as both the counter and working electrode and a slice of Celgard 2400 separator soaked with electrolytes. The electrolytes used for the lithium/lithium symmetric test were saturated with SO_2_ gas by bubbling in prepared electrolytes. Electrochemical impedance measurements were performed by using a potentio-galvanostat (VSP-300, Bio-Logic Science Instruments, France) at room temperature with a frequency range from 200 kHz to 50 mHz. A Bruker D2-Phaser with Cu-Kα radiation (γ=1.5406 Å) was used to obtain XRD spectra of the cathodes under an Ar atmosphere with an air-locking holder. The morphology of the products in the electrode was examined using FE-SEM (MERLIN Compact; Zeiss, Germany) after platinum coating. XPS (Thermo VG Scientific, Sigma Probe, UK) was used for the surface characterization of the cathodes in a argon atmosphere without air exposure. For the *in situ* gas analyses, a DEMS instrument constructed with the combination of a mass spectrometer (MS; HPR-20, Hiden Analytical) and potentiogalvanostat was used, as described in our previous report.^12^ *In situ* gas analyses were conducted using argon carrier gas at a constant flow rate of 10 ml min^−1^ during the charge process after the full relaxation of the DEMS cell. Dielectric constants of prepared solutions were measured at 20 °C by using Liquid Dielectric Constant Meter (Model 871; Nihon Rufuto, Japan). Ultraviolet–visible spectroscopy (Cary 5000; Agilent, USA) was used for SO_2_ solution characterizations.

### Data availability

The data that support the findings of this study are available from the authors on reasonable request.

## Additional information

**How to cite this article:** Park, H. *et al*. High-efficiency and high-power rechargeable lithium–sulfur dioxide batteries exploiting conventional carbonate-based electrolytes. *Nat. Commun.* **8,** 14989 doi: 10.1038/ncomms14989 (2017).

**Publisher's note**: Springer Nature remains neutral with regard to jurisdictional claims in published maps and institutional affiliations.

## Supplementary Material

Supplementary Information Supplementary Figures, Supplementary Tables, Supplementary Note and Supplementary References
