# Supplementary material for: High-efficiency and high-power rechargeable lithium–sulfur dioxide batteries exploiting conventional carbonate-based electrolytes
Source: Nat Commun. 2017 May 11;8:14989. doi: 10.1038/ncomms14989 (PMC5437267; doi:10.1038/ncomms14989)
Supplement: Supplementary Information — Supplementary Figures, Supplementary Tables, Supplementary Note and Supplementary References [file ncomms14989-s1.pdf]

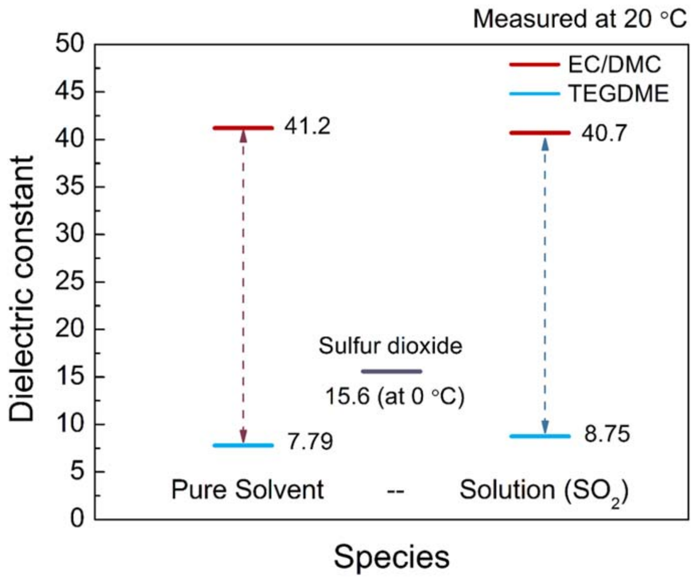

Supplementary Figure 1 | Measurements of the dielectric constants of organic solvents with saturation of sulfur dioxide. Organic solutions containing sulfur dioxide were prepared through a simple saturation of sulfur dioxide gas to the organic solvents in a confined chamber under the pressure of 1 bar. Dielectric constants of prepared solutions were measured at 20 °C by using Liquid Dielectric Constant Meter (Model 871, Nihon Rufuto, Japan).

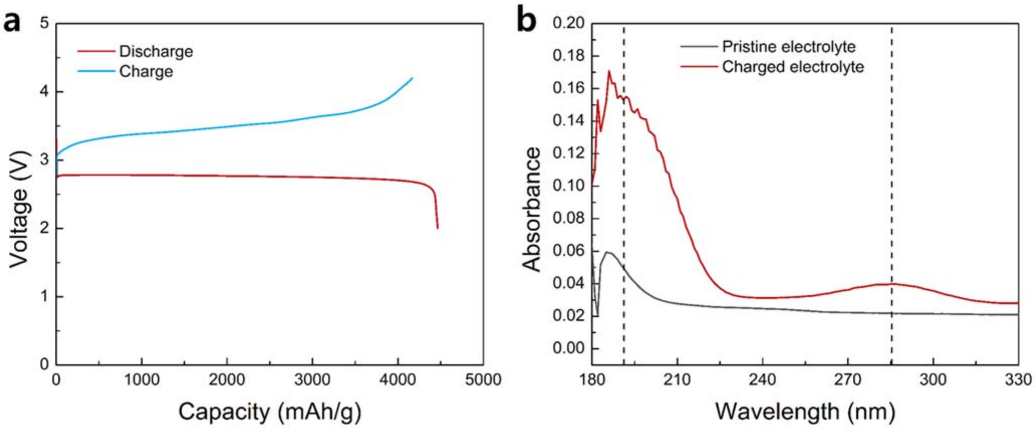

\

Supplementary Figure 2 | Reversible SO_2 evolution in the electrolyte solutions. a, Discharge profile of closed-type lithium-sulfur dioxide cells by using SO_2 saturated EC/DMC electrolyte (Red) and charge profile of rebuilt cell with fresh electrolyte and pre-discharged gas electrode (Blue). **b**, UV-vis spectra of the electrolyte solution after charging the pre-discharged gas-electrode

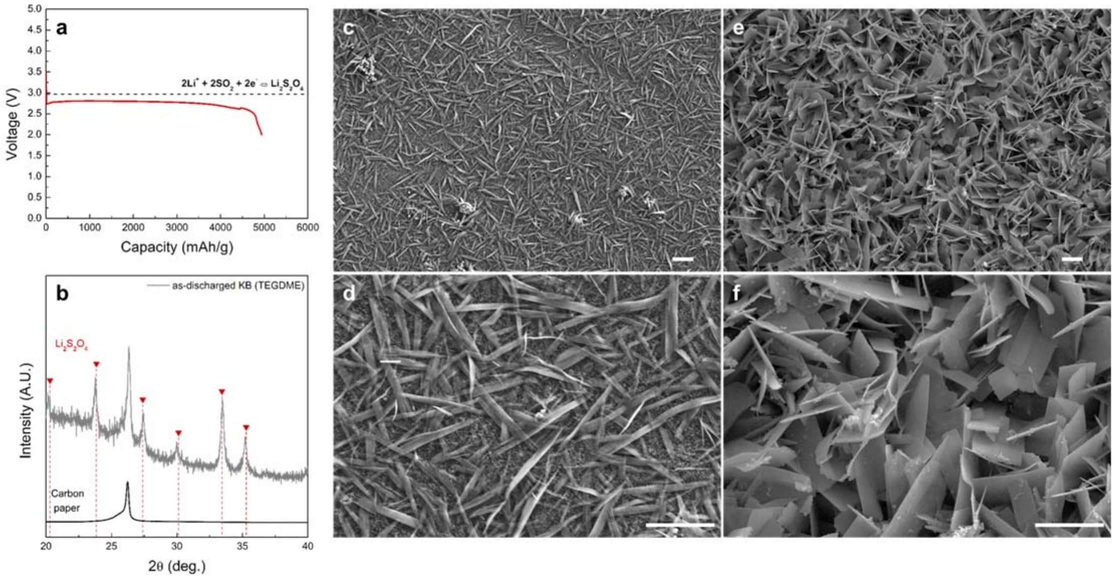

Supplementary Figure 3 | Comparison of morphological evolution of discharge products under carbonate and ether electrolytes. a, Discharge profiles of lithium–sulfur dioxide cells with TEGDME electrolyte. **b**, XRD pattern of discharged electrode of lithium–sulfur dioxide cells. (**c–f**) SEM images of discharge products on the carbon cathodes with (**c,d**) ether electrolyte and (**e,f**) carbonate electrolyte. (scale bar 2 μm)

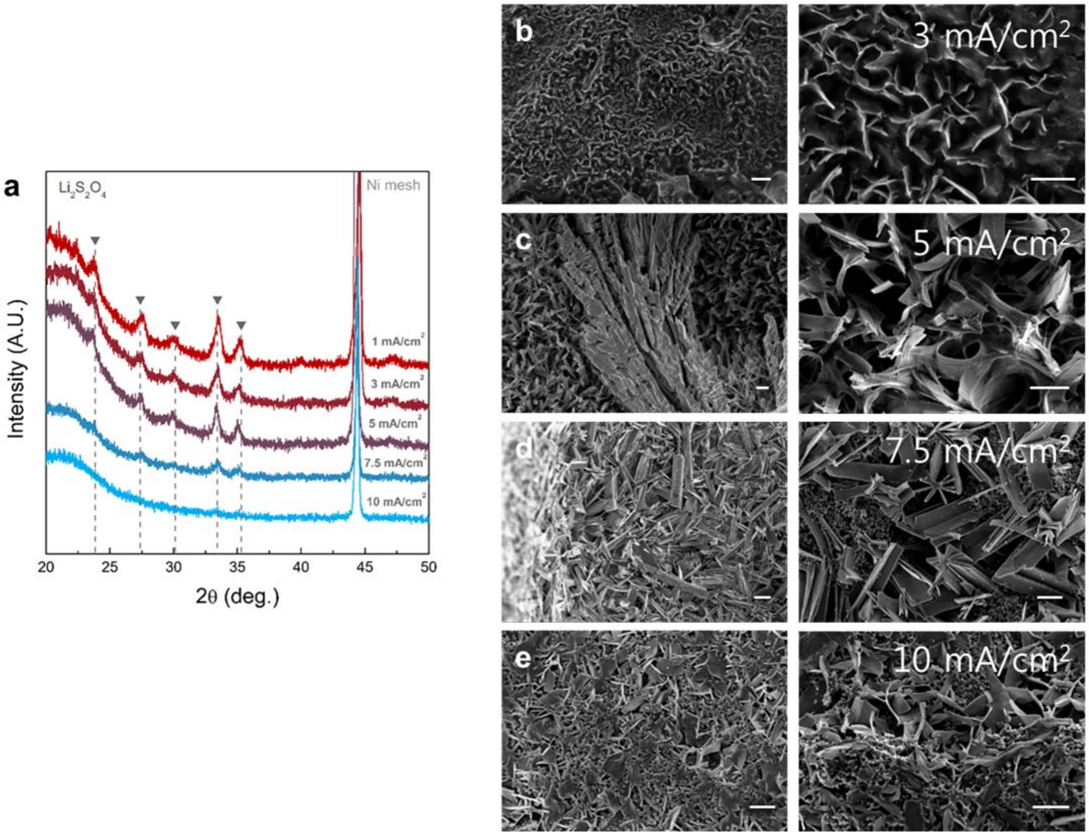

Supplementary Figure 4 | Identification of discharge products and corresponding morphological observations. a, XRD patterns of discharged cathode of lithium–sulfur dioxide cells with different current densities. The high-intensity peaks at 44° correspond to the Ni mesh current collector. Ni mesh was used particularly for the rate capability tests to achieve a high electric conductivity for the current collecting substrate when applying a high current density. (**b–e**), Corresponding SEM images of discharged cathodes at (**b**) 3 mA/cm^2, (**c**) 5 mA/cm^2, (**d**) 7.5 mA/cm^2, and (**e**) 10 mA/cm^2. (scale bar 2 μm (left), 1 μm (right))

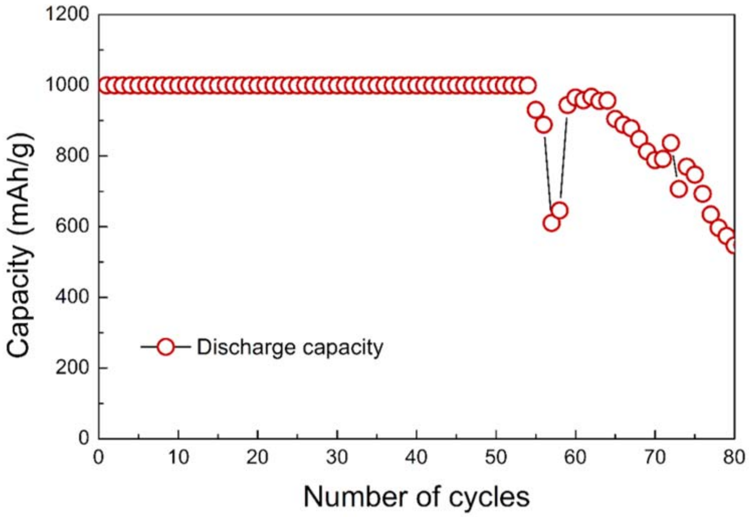

Supplementary Figure 5 | Cycle properties under a capacity of 1,000 mAh/g of lithium-sulfur dioxide batteries with carbonate-based electrolytes.

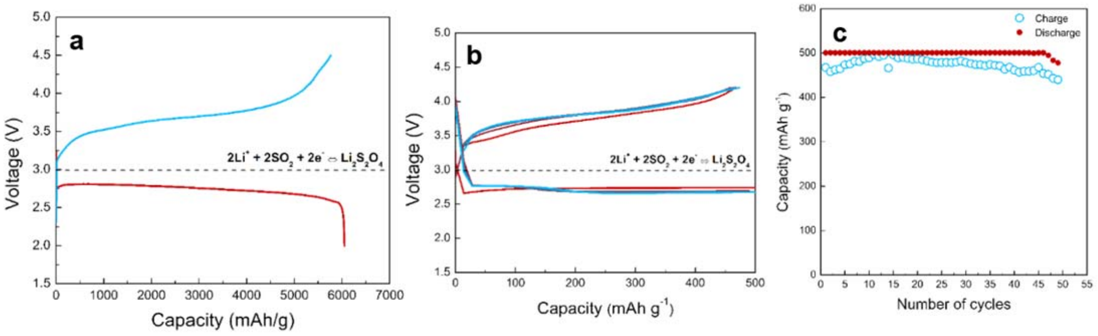

Supplementary Figure 6 | Electrochemical properties of lithium–sulfur dioxide cells with carbonate electrolyte at high current density of 1 mA/cm^2. **a,** Galvanostatic discharge/charge profiles of lithium–sulfur dioxide cells at 1 mA/cm^2 with voltage cut-offs of 2 and 4.5 V. **b**, Electrochemical profiles of lithium–sulfur dioxide cells at 1 mA/cm^2 with capacity cut-off of 500 mAh/g for 5 cycles. **c**, Corresponding cyclability of lithium–sulfur dioxide cells.

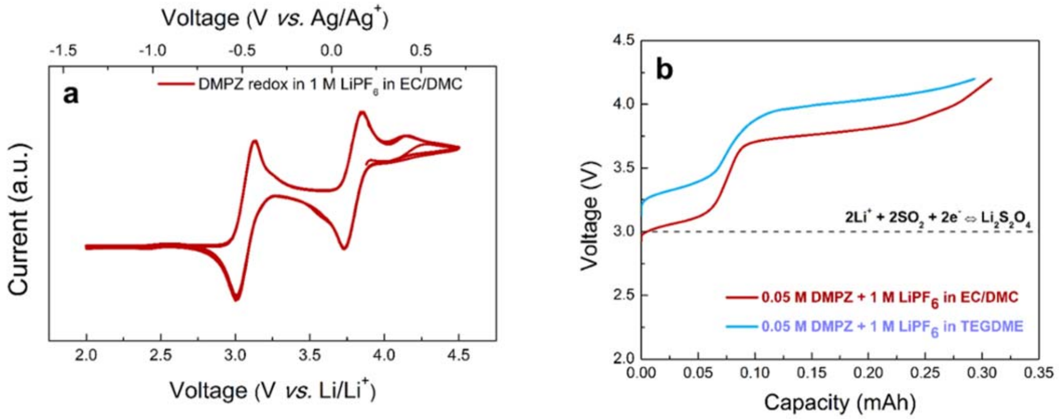

Supplementary Figure 7 | Redox potentials of DMPZ soluble catalyst under different electrolytes. a, Cycle voltammogram of 50 mM DMPZ dissolved in 1 M $LiPF_6$ in EC/DMC at a scan rate of 100 mV/s with 3-electrode cell configuration. (working electrode: gold; counter electrode: platinum; reference electrode: 0.05 M Ag/$AgNO_3$ in acetonitrile) **b**, Galvanostatic charge profiles of 50 mM DMPZ dissolved in 1 M $LiPF_6$ in EC/DMC and TEGDME at 0.2 mA/cm^2 under Ar atmosphere with 2-electrode cell configuration.

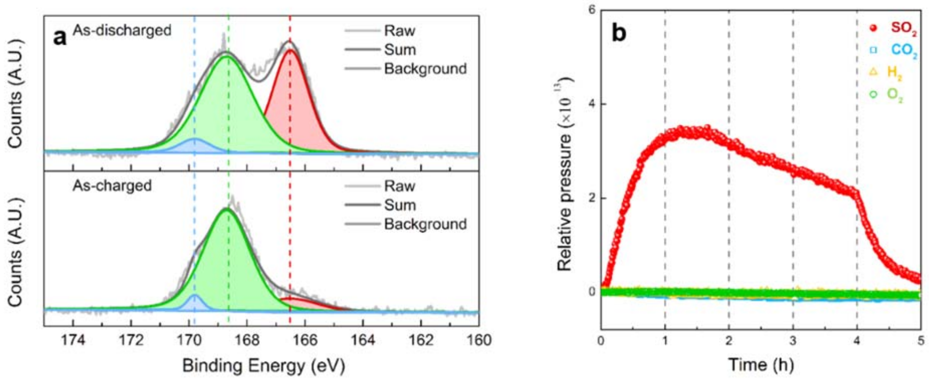

Supplementary Figure 8 | Electrochemical activity of DMPZ soluble catalyst in lithium–sulfur dioxide cells with carbonate electrolyte. a, *Ex situ* XPS results of discharged and charged cathodes for lithium–sulfur dioxide cells with DMPZ catalyst. **b**, *In situ* gas analysis results of lithium–sulfur dioxide cells with DMPZ catalyst during charge through DEMS.

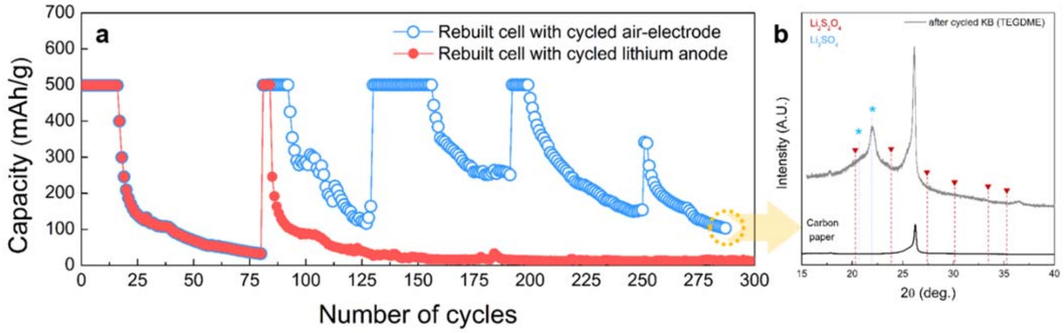

	Initial	1st rebuilt	2nd rebuilt	3rd rebuilt	4th rebuilt	Summation
**Cyclability**	16	12	27	8	0	63

Supplementary Figure 9 | Cycle capability of carbon cathodes for lithium–sulfur dioxide cell with ether electrolyte. a, Cycle properties of continuously rebuilt lithium–sulfur dioxide cells with fresh Li anode after cycling: **b**, XRD pattern of cathodes after cycling of lithium–sulfur dioxide cell.

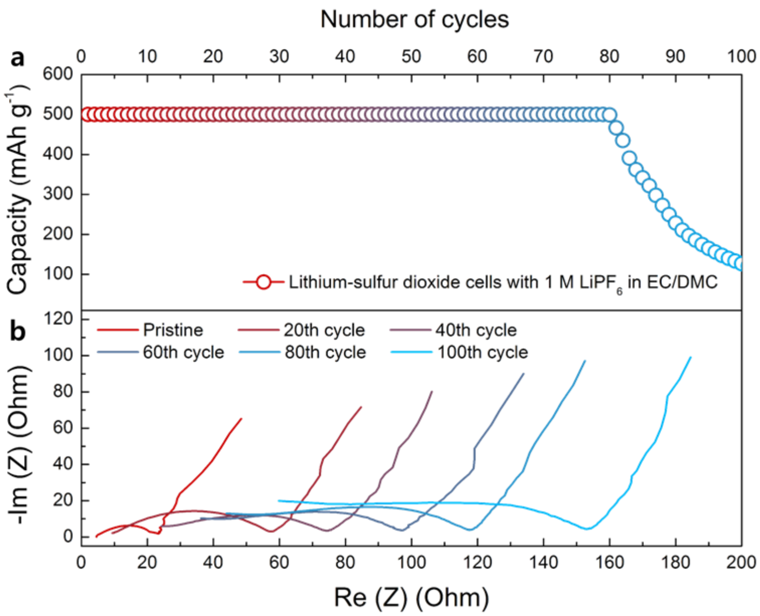

Supplementary Figure 10 | Electrochemical impedance spectroscopy results of lithium-sulfur dioxide cells with cycling. a, Cyclability of lithium-sulfur dioxide cells under a limited capacity of 500 mAh/g. **b**, Nyquist plots for the impedances of the cell with cycling.

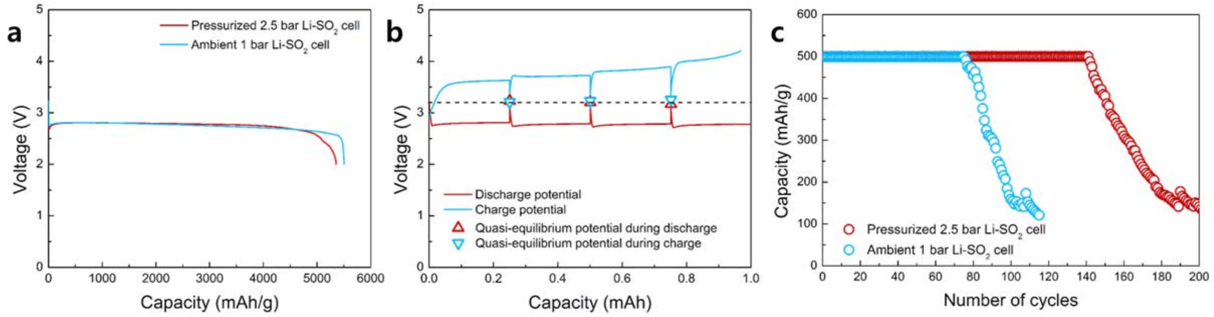

Supplementary Figure 11 | Electrochemical properties of pressurized lithium-sulfur dioxide cells. a, Discharge profiles for the 1st cycle of the ambient pressure cell and pressurized cell. **b**, GITT analysis result of pressurized cells. **c**, Cyclability of the ambient pressure cells and pressurized cells.

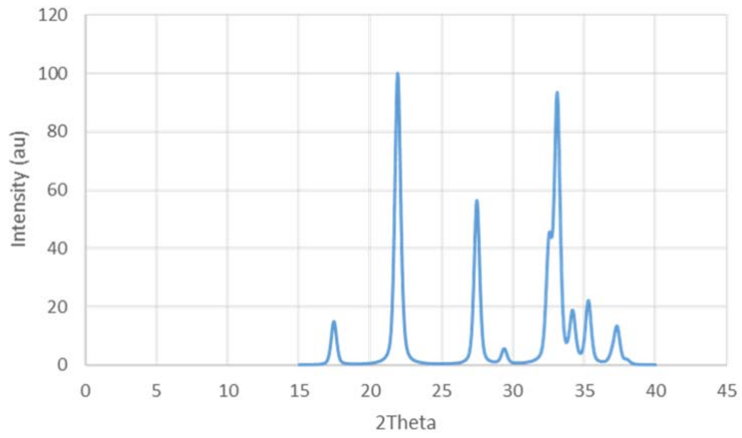

Supplementary Figure 12 | Simulated XRD pattern of theoretically optimized $Li_2S_2O_4$ crystal structure based on $Na_2S_2O_4$. The crystal structure for $Li_2S_2O_4$ was hypothetically generated by substituting Na atoms with Li atoms based on the $Na_2S_2O_4$ structure because there is no experimentally determined crystal structure (only available powder diffraction peak data, JCPDS #36-1101). The simulated XRD pattern and theoretical reaction potential (Table S2) indicate that the hypothetical $Li_2S_2O_4$ crystal structure was reasonably selected.

Supplementary Table 1 | Elementary reaction steps with corresponding energy changes under carbonate and ether electrolyte.

a

EC/DMC	Reaction	ΔE (eV)
C-1	$2SO_2(g) + 2e^- \rightarrow 2SO_2^-(soln)$	-4.70 x 2
C-2	$2SO_2^-(soln) \rightarrow S_2O_4^{2-}(soln)$	-0.23
C-3	$S_2O_4^{2-}(soln) + Li^+(soln) \rightarrow LiS_2O_4^-(soln)$	+0.63
C-4	$LiS_2O_4^-(soln) + Li^+(soln) \rightarrow Li_2S_2O_4(soln)$	+0.78
C-5	$Li_2S_2O_4(soln) \rightarrow Li_2S_2O_4(s)$	-1.45
A-1	$2Li(s) \rightarrow 2Li^+(soln) + 2e^-$	+1.60 x 2
**Total**	$\mathbf{2SO_2(g) + 2Li(s) \rightarrow Li_2S_2O_4(s)}$	**-3.24 x 2**

b

TEGDME	Reaction	ΔE (eV)
C-1	$2SO_2(g) + 2e^- \rightarrow 2SO_2^-(soln)$	-4.40 x 2
C-2	$SO_2^-(soln) + Li^+(soln) \rightarrow LiSO_2(soln)$	+0.07
C-3	$LiSO_2(soln) + SO_2^-(soln) \rightarrow LiS_2O_4^-(soln)$	-0.67
C-4	$LiS_2O_4^-(soln)\ Li^+(soln) \rightarrow Li_2S_2O_4(soln)$	+0.04
C-5	$Li_2S_2O_4(soln) \rightarrow Li_2S_2O_4(s)$	-1.39
A-1	$2Li(s) \rightarrow 2Li^+ + 2e^-$	+2.22 x 2
**Total**	$\mathbf{2SO_2(g) + 2Li(s) \rightarrow Li_2S_2O_4(s)}$	**-3.16 x 2**

Supplementary Table 2 | Measured ionic conductivities of carbonate and ether electrolytes with and without saturated SO_2 gas.

σ_{ion} [mS cm^{-1}]	Without SO_2	SO_2 bubbled	Reference
1M $LiPF_6$ in EC/DMC	11.35 ± 0.37	11.84 ± 0.26	11.7 [1]
1M LiTFSi in TEGDME	2.91 ± 0.04	3.35 ± 0.01	2.72 [2]
1M $LiPF_6$ in TEGDME	2.33 ± 0.01	2.70 ± 0.05	1.86 [3]

Supplementary Table 3 | Theoretical reaction energy changes for possible formation pathways of byproduct Li_2SO_4 in lithium–sulfur dioxide cell atmosphere.

**Li_2SO_4**	Reaction	ΔE
**(1)**	$2Li^+ + SO_4^{2-} \rightarrow Li_2SO_4$ (s)	-0.02 eV
**(2)**	$Li_2S_2O_4$ (s) $\rightarrow Li_2SO_4$ (s) + 1/8 S_8	-1.18 eV

Supplementary Table 4 | Parameters for Poisson–Boltzmann implicit solvation calculation. The dielectric constant (ε) and probe radii (R) for the electrolyte solvents were determined based on experimental literature values.[4]

Solvent	Dielectric constant, ε	Molar mass, Mw (g/mol)	Density, ρ (g/cc)	Probe radius, R (Å)
Ethylene carbonate (EC)	95.3	88.06	1.32	2.36
Dimethyl carbonate (DMC)	3.1	90.08	1.07	2.56
EC/DMC (1:1 vol%)	35.0	88.95	1.20	2.45
Tetraethylene glycol dimethyl ether (TEGDME)	7.8	222.28	1.01	3.52

Supplementary Note 1 (Supplementary Figure 11): We constructed the lithium–sulfur dioxide cells confined with high-pressure sulfur dioxide gas and examined the electrochemical performances of the cell by means of full discharge, GITT, and cycle tests. The cell with the pressure of 2.5 bar was investigated, which is the maximum pressure achievable in our lab-scale pressurized cell setup. Excessive pressurization with sulfur dioxide was avoided due to the safety concerns in this study. Supplementary Figure 11a presents the discharge profile for the 1st cycle of the pressurized cell, showing that almost identical discharge capacity and voltage were obtained to those of the ambient pressure cell. The invariance of the discharge capacity regardless of the gas pressure is attributed to fact that the deliverable capacities of the current lithium–sulfur dioxide cells are limited by the gas-electrode surface area where the full accommodation of discharge products induces the rapid increase in the overpotential. In order to more closely examine the reaction voltage of the pressurized cells, GITT analysis was carried out to measure the quasi-equilibrium potentials of the cells. As shown in Supplementary Figure 11b, a slightly increased open circuit potential of 3.2 V (vs. Li/Li^+) was obtained in the pressurized cell compared with the value of 3.0 V (vs. Li/Li^+) for ambient pressure cell presented in the Figure 2b of the manuscript. It is quite reasonable in thermodynamic aspects that the high activity of the gas from the increased pressure might result in a more negative value of the Gibbs free energy change of the total reaction, leading to an increase in the open-circuit potential of the pressurized cells. However, the simple estimation by using Nernst equation ($\Delta E = \frac{RT}{nF} ln\, a_{SO_2} = 0.0235\,V$, where n equals to the number of electron transferred, F equals to Faraday constant, R equals to gas constant, T equals to the temperature, and a equals to the activity) shows only 0.02V increase and cannot fully explain the increase of about 0.2 V, which has to be further studied with the careful consideration of total energies of

other reactants and products involved in the reactions.

Additionally, we examined the cyclability of the pressurized lithium–sulfur dioxide cells under a limited capacity mode in Supplementary Figure 11c. Interestingly, the pressurized cell could exhibit better cycling behavior about 140 times compared to the number of cycles achievable in the ambient-pressure cell. Considering that the thermodynamic stability of $Li_2S_2O_4$ discharge products have a vital role in the formation and accumulation of inactive byproducts including Li_2SO_4 as discussed in the manuscript, it is our speculation that the high pressure atmosphere might have a positive impact on the thermodynamic stability of $Li_2S_2O_4$, probably leading to the enhancement of cycle stability by suppressing the self-decomposition of $Li_2S_2O_4$. Even though the precise origin of the improved electrochemical properties is not fully addressed at the current stage, it has to be further investigated in the following study, and the pressure control of the cell might be one of the possible strategies toward the highly reversible lithium–sulfur dioxide batteries.

Supplementary References

1 Xu, K. Electrolytes and Interphases in Li-Ion Batteries and Beyond. *Chem. Rev.* **114**, 11503-11618 (2014).

2 Park, M. S. *et al.* A highly reversible lithium metal anode. *Sci. Rep.* **4**, 3815 (2014).

3 Choi, J. W. *et al.* Effect of various lithium salts in TEGDME based electrolyte for Li/pyrite battery. *Solid State Phenom.* **124-126**, 971-974 (2007).

4 Hall, D. S., Self, J. & Dahn, J. R. Dielectric constants for quantum chemistry and Li-ion batteries: solvent blends of ethylene carbonate and ethyl methyl carbonate. *J. Phys. Chem. C* **119**, 22322-22330 (2015).
